# GDF15 as a potential biomarker to distinguish fibrotic from non-fibrotic hypersensitivity pneumonitis

**DOI:** 10.1038/s41598-023-49459-6

**Published:** 2024-01-09

**Authors:** A. Alarcon-Dionet, A. Ruiz, L. Chavez-Galan, I. Buendia-Roldan, M. Selman

**Affiliations:** 1 Translational Research Laboratory on Aging and Pulmonary Fibrosis, Instituto Nacional de Enfermedades Respiratorias Ismael Cosío Villegas, 14080 Mexico City, Mexico; 2 Laboratory of Integrative Immunology, Instituto Nacional de Enfermedades Respiratorias Ismael Cosío Villegas, 14080 Mexico City, Mexico

**Keywords:** Biomarkers, Respiratory tract diseases

## Abstract

Hypersensitivity Pneumonitis (HP) is an immune-mediated interstitial lung disease (ILD) characterized by fibrotic HP (fHP) or non-fibrotic HP (non-fHP). Fibrosis is associated with poor prognosis, emphasizing the need for biomarkers to distinguish fHP from non-fHP. This study aimed to determine the plasma levels of GDF15 in HP patients and assess its association with lung function and phenotype classification. GDF15 levels were quantified by ELISA in HP (n = 64), idiopathic pulmonary fibrosis (n = 54), and healthy control (n = 128) groups. Clinical, demographic, and functional data were obtained from medical records. High-resolution chest CT scans were used to classify HP patients into fHP and non-fHP groups. In addition, receiver operating characteristic analysis was performed to determine the cut-off point, sensitivity, and specificity. Our results revealed significantly elevated GDF15 levels in fHP compared to non-fHP (2539 ± 821 pg/ml versus 1783 ± 801 pg/ml; p = 0.009). The estimated cut-off point for plasma GDF15 levels to distinguish fHP from non-fHP was 2193.4 pg/ml (AUC 0.75). These findings suggest that GDF15 may serve as a valuable biomarker for differentiating between fHP and non-fHP, potentially indicating its involvement in lung fibrosis development in HP.

## Introduction

Hypersensitivity Pneumonitis (HP) is a complex immune-mediated interstitial lung disease (ILD) that occurs in susceptible individuals following exposure to known or unknown antigens. The presence of radiographic or histopathological fibrosis is associated with a poor prognosis, leading to the recent categorization of HP based on the predominance of fibrosis or inflammation as fibrotic HP (fHP) or non-fibrotic HP (non-fHP), respectively^1^. However, there is currently a lack or identified biomarkers to differentiate between fHP and non-fHP.

Growth and differentiation factor 15 (GDF15) is a divergent protein belonging to the transforming growth factor beta (TGFβ) superfamily and is part of the senescence-associated secretory phenotype (SASP) protein repertoire^2–4^.

GDF15 has been implicated in various physiological and pathological conditions, including inflammation, aging, smoking, cancer, and hypoxia^3,4^. In the context of the lungs, GDF15 has been associated with disease severity, exacerbation, prognosis, and decline of lung function in chronic obstructive pulmonary disease (COPD)^3,5^. Additionally, in patients with Systemic Sclerosis, serum GDF15 levels are correlate with disease activity, ILD, and declining lung function^6–8^.

Similary, in idiopathic pulmonary fibrosis (IPF), an aggressive ILD associated with aging, GDF15 expression has been linked to disease severity, exacerbation, and prognosis^9–11^.

Notably, there are several clinical and pathogenic similarities between IPF and fHP, including disease progression, transcriptomic profile, and an increased frequency of rare and common gene variants such as MUC5B and variants associated with telomere biology^12–14^. However, no studies have explored the circulating levels of GDF15 in HP.

Therefore, considering the implication of GDF15 in lung fibrosis, we hypothesized that elevated baseline plasma concentrations of GDF15 could serve as a differentiating factor between fHP and non-fHP phenotypes. Thus, this study aimed to determine the plasma levels of GDF15 in HP and investigate its association with lung function and the fibrotic (fHP) and non-fibrotic (non-fHP) phenotypes.

## Results

### Baseline demographics and functional characteristics of patients with HP, IPF and controls

A total of 246 patients were included in the study, consisting of 64 HP patients, 54 IPF patients, and 128 healthy controls (HC) (Table 1). Patients with HP were younger, predominantly female, and non-smokers. Pulmonary function tests alterations were similar between patients with HP and IPF, as shown in Table 1.

**Table 1:** Baseline demographics and functional characteristics of the 3 groups.

	HC n = 128	HP n = 64	IPF n = 54	p
Age (SD)	68 ± 8	53 ± 10	65 ± 7	< 0.001
Female n (%)	76 (59)	57 (89)	4 (7)	< 0.001
Smoking n (%)	63 (49)	16 (25)	38 (70)	< 0.001
FVC L (SD)	2.9 ± 1	1.8 ± 1	2.3 ± 1	< 0.001
FVC %pred (SD)	93 ± 16	61 ± 18	67 ± 18	< 0.001
DLCO %pred (SD)	111 ± 23	56 ± 21	52 ± 19	< 0.001
6MWD meters (SD)	447 ± 111	385 ± 123	375 ± 140	< 0.001
SpO2 basal (SD)	94 ± 2	93 ± 3	92 ± 3	0.1
SpO2 final (SD)	92 ± 5	84 ± 7	79 ± 7	< 0.001
PASP (SD)	NA	33 ± 7	41 ± 20	0.2

Significant values are in bold.

*HC* healthy controls, *HP* hypersensitivity pneumonitis, *IPF* idiopathic pulmonary fibrosis, *FVC* forced vital capacity, *DLCO* diffusing capacity for carbon monoxide, *6-MWD* 6-min walk distance, *SpO2* percutaneous arterial oxygen saturation, *PASP* pulmonary artery systolic pressure.

At the time of sample collection, no patient had received treatment.

Upon classifying HP patients into fibrotic and non-fibrotic, we observed that fHP patients were older and exhibited worse pulmonary function in terms of forced vital capacity [FVC] (57 ± 17% versus 65 ± 18%, p = 0.02) and adjusted diffusing capacity for carbon monoxide [DLCO] (10.2 ± 4 ml/mmHg versus 13.5 ± 6 ml/mmHg, p = 0.04) (Table 2). Additionally, fHP patients had a significantly lower percentage of lymphocytes in bronchoalveolar lavage (BAL) fluids. No significant differences were found in the time of symptoms before diagnosis, smoking habits, or identification of the offending antigen.

**Table 2:** Baseline demographics, clinical, and functional characteristics fibrotic and non-fibrotic HP.

	Non-fibrotic HP n = 34	Fibrotic HP n = 30	p
Age (SD)	50 ± 10	56 ± 10	0.02
Female n (%)	32 (94%)	25 (83%)	0.2
Smoking n (%)	8 (23%)	8 (26%)	0.7
Antigen exposure			
Avian n (%)	19 (55)	15 (50)	
Fungi n (%)	1 (3)	1 (3)	0.9
Others n (%)	NA	1 (3)	
Non identified n (%)	14 (42)	13 (44)	
Time to diagnostic months (SD)	26 ± 30	24 ± 27	0.8
FVC % pred (SD)	65 ± 18	57 ± 17	0.02
DLCO ml/min/mmHg (SD)	13.5 ± 6	10.2 ± 4	0.04
DLCO % pred (SD)	59 ± 20	52 ± 21	0.1
6MWD meters (SD)	397 ± 129	372 ± 129	0.2
SpO2 basal (SD)	93 ± 4	93 ± 3	0.8
SpO2 final (SD)	85 ± 8	83 ± 6	0.2
PASP (SD)	30 ± 5	35 ± 8	0.4
BAL	n = 33	n = 27	
% Macrophages (SD)	44 ± 21	56 ± 21	0.04
% Lymphocytes (SD)	54 ± 22	41 ± 20	0.02
GDF15 plasma concentration pg/ml (SD)	1783 ± 801	2539 ± 821	0.009
GDF15 BAL fluid concentration pg/ml (SD)	183 ± 149	128 ± 77	0.49

Significant values are in bold.

*HP* hypersensitivity pneumonitis, *FVC* forced vital capacity, *DLCO* diffusing capacity for carbon monoxide, *6-MWD* 6-min walk distance, *PASP* pulmonary artery systolic pressure, *SpO2* percutaneous arterial oxygen saturation, *BAL* bronchoalveolar lavage.

### Plasma levels of GDF15

**Figure 1:**
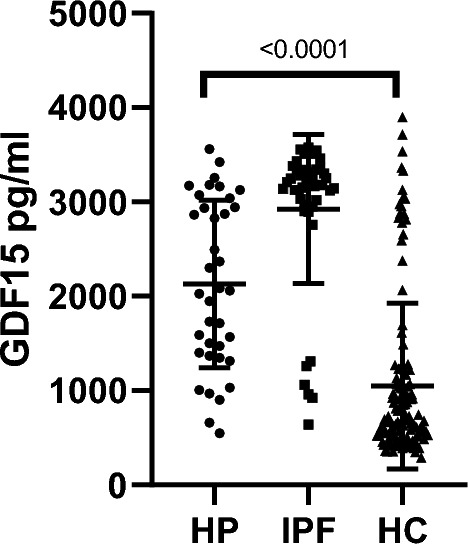
Plasma Levels of GDF15. The plasma levels of GDF15 were measured in patients with Hypersensitivity Pneumonitis (HP), Idiopathic Pulmonary Fibrosis (IPF), and healthy controls (HC), p < 0.0001.

Plasma levels of GDF15 were significantly higher in HP patients compared to HC (2131 ± 886 pg/ml versus 1047.8 ± 877 pg/ml; p < 0.0001) although they did not reach the levels observed in IPF patients (2923 ± 790 pg/ml) (Fig. 1).

**Figure 2:**
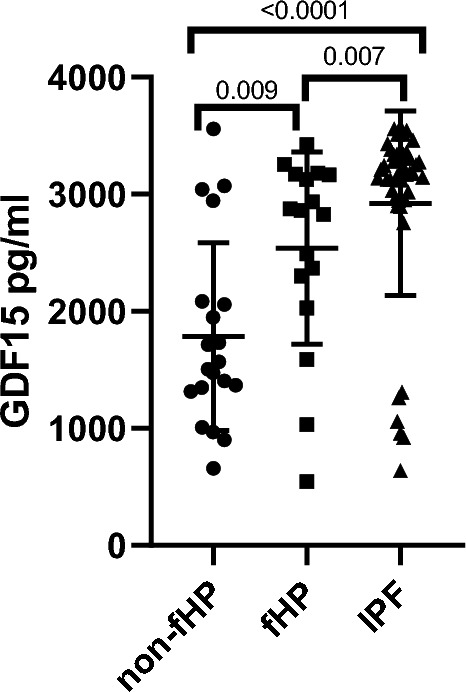
Plasma Levels of GDF15 in IPF, fHP and non-fHP. Compared the plasma levels of GDF15 among patients with Idiopathic Pulmonary Fibrosis (IPF), fibrotic Hypersensitivity Pneumonitis (fHP), and non-fibrotic Hypersensitivity Pneumonitis (non-fHP).

When HP patients were sub-grouped based on their high-resolution computed tomography (HRCT) patterns, the fHP group exhibited markedly higher plasma levels of GDF15 compared to the non-fHP group (2539 ± 821 pg/ml versus 1783 ± 801 pg/ml (p = 0.009) (Fig. 2).

**Figure 3:**
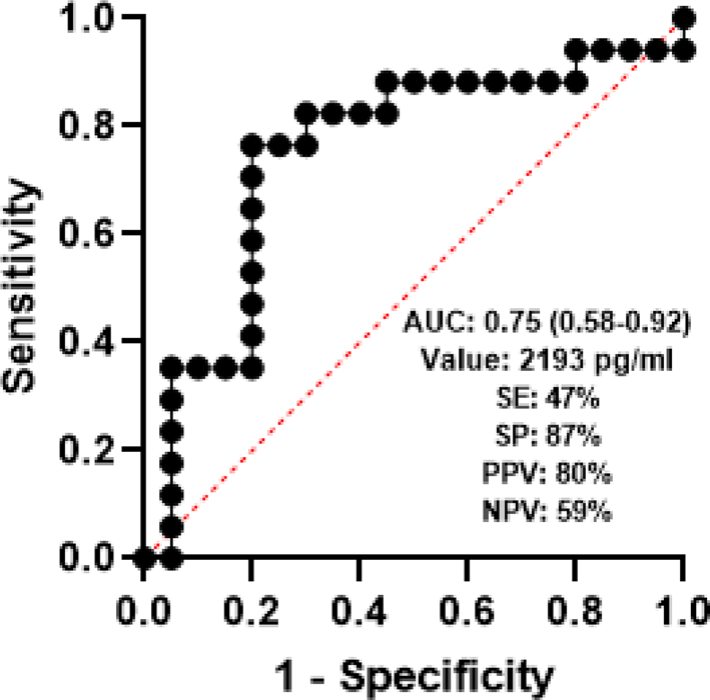
ROC curve for HP fibrotic diagnostic. *ROC* receiver-operating characteristic, *HP* hypersensitivity pneumonitis, *AUC* area under the curve, *SE* sensitivity, *SP* specificity, *PPV* positive predictive value, *NPV* negative predictive value.

To determine the diagnostic accuracy of plasma GDF15 levels in distinguishing between fHP and non-fHP, we performed a Receiver Operating Characteristic (ROC) curve analysis. The area under the curve (AUC) was 0.75 (95% CI: 0.58–0.92) indicating moderate accuracy in differentiating the two phenotypes. Furthermore, the optimal cut-off value for plasma GDF15 levels was 2193.4 pg/ml with a sensitivity of 47% and a specificity of 87% (Fig. 3).

### BAL levels of GDF15

The concentration of GDF15 in BAL fluids was markedly lower than in plasma, and no differences were observed between fibrotic HP and non-fibrotic HP (Table 2).

### Plasma GDF15 and lung function

**Figure 4:**
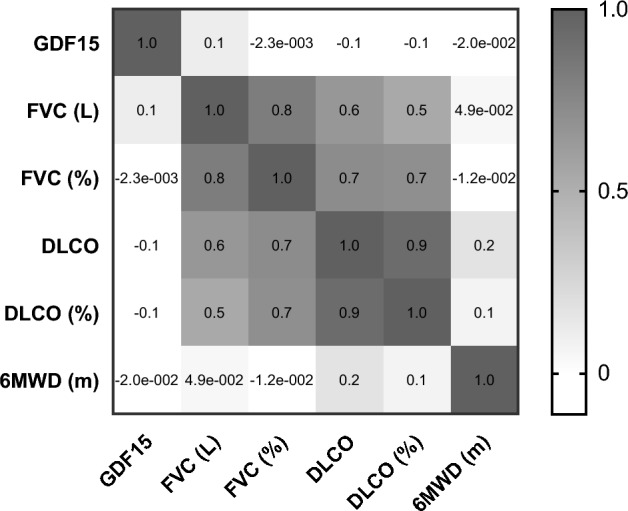
Correlogram between lung functional test and GDF15 plasma levels. Correlations between GDF15 and critical lung function parameters, including Forced Vital Capacity (FVC), Diffusing Capacity for Carbon Monoxide (DLCO), and the 6-min walk distance (6MWD). GDF15 is an abbreviation for Growth Differentiation Factor 15.

We examined the association between GDF15 plasma levels and baseline pulmonary function indexes, including Forced Vital Capacity (FVC), Diffusing Capacity for Carbon Monoxide (DLCO), and the 6-min walk distance (6MWD) (Fig. 4).

## Discussion

A substantial body of evidence indicates that fibrotic HP has a poor prognosis comparable to IPF, which is considered the most aggressive ILD^16,17^.

However, there is a significant lack of reliable biomarkers that can differentiate fHP from non-fHP, highlighting the need for prognostic biomarkers^18^.

GDF-15 has been proposed as a potential biomarker in several chronic respiratory disorders, including COPD^5^, IPF^6^, and ILD associated with systemic sclerosis, particularly those cases with lung fibrosis^10,11^.

In our study, we demonstrated by the first time, that the levels of circulating GDF15 were increased in HP compared with controls, but more importantly, this increase was mostly related to the presence of lung fibrosis. Therefore, when HP was grouped according to its tomographic pattern in fibrotic and non-fibrotic, the plasma levels of GDF15 were markedly higher in fHP. Moreover, our findings indicate that a cut point of 2193 pg/ml is suggestive for fibrotic HP; however, the relatively low sensitivity (although high specificity) of the ROC analysis, preclude to recommend the use of antifibrotic drugs, and patients should be followed treated with immunosuppressive drugs to determine progression, before indicating antifibrotic treatment.

Interestingly, the levels of GDF15 in BAL fluids were strikingly lower than in plasma and showed no differences between fibrotic and non-fibrotic HP.

The exact contribution of GDF15 to the development of fibrosis remains unclear. Notably, GDF15 has been associated with cell senescence, a hallmark of the aging process. It is highly secreted by fibroblasts and epithelial cells as part of the secretory SASP^18^. It has been also implicated as a potential causal link with age-related decline in immune function^19^.

In this context, epithelial cell senescence has been reported in IPF and fHP, mainly those with usual interstitial pneumonia (UIP)-like lesions^20,21^. Moreover, single-cell RNA sequencing has identified the existence of an aberrant type of a strongly profibrotic senescent epithelial cells, located at the edge of myofibroblast foci in the IPF lungs. These cells were also identified in other fibrotic ILD, including fHP^22–24^.

However, while in IPF high levels of GDF15 correlated with the reduction of pulmonary function^6^, in our study we did not observe any correlation with FVC, DLCO, and 6-MWD, probably because the small number of patients analyzed.

Actually, this a limitation of our study, the sample size was relatively small, and since the short time of follow-up, the relationship between GDF15 levels and survival was not evaluated.

In summary, our study provides novel findings by demonstrating increased circulating levels of GDF15 in patients with HP compared to healthy controls, primarily driven by fibrosis. GDF-15 may serve a reliable marker for the detecting fibrosis in HP and could be a potential therapeutic target for the treating pulmonary fibrosis.

## Methods

### Study population

This study was performed in a cohort of patients with HP and IPF at the National Institute of Respiratory Diseases Ismael Cosio Villegas (INER). Clinical data were extracted from medical records, and included baseline demographics, pulmonary function tests, and HRCT pattern. Patients were diagnosed with IPF according to criteria established by the American Thoracic Society/European Respiratory Society/ Japanese Respiratory Society/Asociacion Latinoamericana de Torax (ATS/ERS/JRS/ALAT)^15^. The diagnosis of fHP and non-fHP was based on the current ATS/JRS/ALAT guidelines and performed by a multidisciplinary team^1^. As controls, healthy subjects of our aging cohort program were evaluated. This study was approved by the INER Ethical and Research committees, (code C30-22), and all participants signed informed consent. All methods were performed in accordance with the relevant guidelines and regulations.

### Pulmonary assessment

Baseline forced vital capacity (FVC), forced expiratory volume in 1 (FEV1), diffusion capacity for carbon monoxide (DLCO) and the walking meters of the 6-min walking test (6-MWT), were obtained from the clinical records. High-resolution CT (HRCT) of the chest was reviewed by a lung radiology expert in ILD and two pulmonologists to determine the HP fibrotic pattern. These patients were classified according to the guidelines recommended by ATS/JRS/ALAT as either fibrotic HP or non-fibrotic HP^1^

### Bronchoalveolar lavage (BAL)

BAL was performed as described^25^. Briefly, BAL was performed through flexible fiberoptic bronchoscopy under local anesthesia and 300 ml of normal saline were instilled in 50-ml aliquots, with an average recovery of 60–70%. The recovered fluid was centrifuged at 250×*g* for 10 min at 4 °C and the supernatants were kept at − 70 °C until use.

### Quantification of GDF15 in plasma and bronchoalveolar lavage

Baseline plasma levels of GDF15 were quantified using a commercially available enzyme-linked immunosorbent assay (ELISA) kit specific for Human GDF15 (Cat. EHGDF15, Invitrogen, ThermoFisher). The assay was performed by comparing the corresponding standard curve and following the manufacturer’s instructions. Briefly, plasma samples were analyzed in duplicate, and the absorbance of the wells was read at 450 nm using a microplate reader. Then, the mean value of the duplicate wells was calculated and reported in pg/mL as the quantified level of GDF15 in the plasma samples.

### Statistical analysis

Quantitative variables were expressed as mean ± standard deviation (SD), while qualitative variables were reported as frequencies and percentages. The normality of variables were determined with the Kolmogorov–Smirnov test. We used the chi-square test to compare categorical variables between the groups and the Mann–Whitney U and Kruskall-Wallis tests with nonparametric data for two or three groups, respectively. The correlations of the quantitative variables were made using Spearman's coefficient. Using receiver operating characteristic (ROC) analysis, the cut-off point, sensitivity, and specificity were determined. The statistical level of significance for all tests was considered 0.05. Statistical analysis was performed with IBM SPSS Statistics Version 26 software package and graph prism Version 8.

## Data Availability

All data generated or analysed during this study are included in this published article.
